# Preharvest Maize Fungal Microbiome and Mycotoxin Contamination: Case of Zambia’s Different Rainfall Patterns

**DOI:** 10.1128/aem.00078-23

**Published:** 2023-05-31

**Authors:** Bwalya Katati, Pierre Schoenmakers, Henry Njapau, Paul W. Kachapulula, Bas J. Zwaan, Anne D. van Diepeningen, Sijmen E. Schoustra

**Affiliations:** a Laborary of Genetics, Wageningen University and Research, Wageningen, The Netherlands; b Mycotoxicology Laboratory, National Institute for Scientific and Industrial Research, Lusaka, Zambia; c School of Agricultural Sciences, University of Zambia, Lusaka, Zambia; d Business Unit Biointeractions and Plant Health, Wageningen University and Research, Wageningen, The Netherlands.; Michigan State University

**Keywords:** aflatoxin, fumonisin-B1, fungi, ITS1, maize, mycobiome

## Abstract

The preharvest maize mycobiome may be crucial in defining the health of the crop in terms of potential disease burden and mycotoxins. We investigated the preharvest maize mycobiome structure, including the influence of weather patterns, in terms of rainfall intensity, on its composition. In addition, we investigated correlation of genera *Fusarium* and *Aspergillus* with maize fumonisin-B1 and aflatoxin. Forty maize fields from selected districts in the wetter northern (N) and drier southern (S) agroecological zones of Zambia were sampled twice over two seasons (1 and 2). The defined weather variables over the two seasons were low rainfall with dry spell (S1), low rainfall (S2), and high rainfall (N1 and N2). High-throughput DNA amplicon sequencing of internal transcribed spacer 1 (ITS1) was used to determine the mycobiome structure and the composition in relation to rainfall patterns. We detected 61 genera, with *Fusarium* and previously unreported *Sarocladium* in Zambia to have the highest frequency of detection on the maize. There was a significant difference in fungal genera composition between S1 and S2 but no difference between N1 and N2. The weather pattern with dry spell, S1, had a strong proliferation of *Meyerozyma* and xerophiles *Penicillium*, *Kodamaea*, and *Aspergillus*. The four genera drove the difference in composition between S1 and S2 and the significantly higher fungal diversity in S1 compared to N2. Of the mycotoxin-important fungi, dry conditions (S1) were a key driver for proliferation of *Aspergillus*, while *Fusarium* proliferation occurred irrespective of weather patterns. The relative abundance of *Aspergillus* and *Fusarium* resonated with maize aflatoxin and fumonisin-B1 levels, respectively.

**IMPORTANCE** Fungi contaminate various crops worldwide. Maize, an important human staple and livestock cereal, is susceptible to contamination with fungi in the field. Fungi are drivers of plant disease and can compromise yield. Some species of fungi are known to produce chemical compounds (mycotoxins), which are cancer-causing agents in humans and impair livestock productivity. It is important to understand the spectrum of fungi on maize and how weather conditions can impact their abundance. This is because the abundance of fungi in the field can have a bearing on the health of the crop as well as potential for mycotoxins contamination. By understanding the spectrum of the preharvest fungi, it becomes possible to know the key fungi adapted to the maize and subsequently the potential for crop disease as well as mycotoxins contamination. The influence of weather conditions on the spectrum of preharvest fungi on maize has not been fully explored.

## INTRODUCTION

Maize (Zea mays) is an important and widely propagated cereal (1) providing essential calories for both human food and livestock feed worldwide. In Zambia, for example, maize is the principal staple and cash crop (2), and approximately 65% of the arable fields are covered by maize. Annual per capita consumption of maize in Zambia is estimated at 105 kg (3) (https://www.yieldgap.org/zambia).

Unfortunately, maize is often contaminated with an array of fungal genera in the field. *Aspergillus*, *Fusarium*, *Penicillium*,and *Stenocarpella* have a worldwide spread and have been commonly reported as fungi contaminating maize (4–7). The importance of fungal genera to crops is that some species may be agents of plant disease. For example, genera such as *Ustilago* are known for causing smut in maize (8). Furthermore, *Fusarium* and *Stenocarpella*, which have been reported in Zambia (5) and several studies elsewhere, are associated with maize ear rot (9–12). Members of the genus *Fusarium* are well-known pathogens both in the tropics and temperate zones (13) and commonly occur on maize in the field. *Aspergillus* and *Penicillium* are known to be postharvest opportunistic pathogens especially proliferating during storage. The other importance of fungi to crops is that some species of certain genera are also known to produce metabolites termed mycotoxins (14, 15), which are harmful to human health and impair livestock productivity. For example, *Fusarium*, *Aspergillus*, and *Penicillium* are the most infamous producers of the important mycotoxins fumonisin (FB) (*Fusarium*), aflatoxin (AF) (*Aspergillus*), and ochratoxin (OTA) (*Penicillium*) (16–18) as well as other compounds such as cyclopiazonic acid.

Temperature and water availability are important factors in fungal contamination of crops such as maize, wheat, and rice (19). In tropical climates, such as Zambia, the climatic conditions can promote fungal growth and subsequently fungal contamination of crops. In the face of climate change, southern African countries are expected to encounter increased unevenness in the rainfall patterns resulting in higher frequency of dry spells, droughts, and periods of heavy precipitation (20, 21). Dry spells during plant growth may translate into higher chances of contamination of grain in the field with xerophilic and moderately xerophilic fungi including *Aspergillus* and *Penicillium*.

Although studies have indicated *Penicillium*, *Fusarium*, and *Aspergillus* to be the common contaminants of maize, it remains possible that other genera could be of equal importance in the contamination of preharvest maize. The importance would be in terms of their potential plant-disease burden due to their high frequency of contamination of the maize in the field. Furthermore, the maize could be host to less reported or indeed rare genera, which may be of phytosanitary importance in terms of plant disease or mycotoxins. Knowledge of the preharvest maize mycobiome structure is vital, as the structure has a bearing on prevalence and relative abundance of pathogenic and mycotoxigenic fungi. For example, the prevalence of *Ustilago* in the mycobiome may have an impact on plant diseases like smut (8) or maize ear rot (9–11). *Ustilago* is seldom reported in sub-Saharan Africa (SSA) despite such a plant disease threat. Despite the known importance of weather patterns in shaping the fungal contamination of crops, it remains unknown how such patterns may influence the maize mycobiome composition in general.

The two main objectives of this study were as follows. (i) Establish the structure of the preharvest maize mycobiome in Zambia. We anticipate a higher diversity of preharvest maize fungi than previously reported, highly contested by *Fusarium* and *Stenocarpella*, as the commonly reported non-xerophilic field fungi. (ii) Determine the influence of prevailing weather conditions on the preharvest maize mycobiome composition. We hypothesize that the composition of the mycobiome is influenced by weather conditions. In addition to the two objectives, we furthermore investigated (iii) the correlation of *Aspergillus* and *Fusarium*, at genus level, with AF and FB1 contamination in relation to rainfall intensity.

## RESULTS

To map the fungal mycobiome by DNA amplicon sequencing, we used the commonly employed method of targeting the ITS region. Considering the high accuracy of amplification, sequencing, and amplicon sequence variant (ASV) generation in the mock community (see Data Set S5 in the supplemental material), we assumed that all generated taxa in the samples amplified similarly. This is given the fact that the mock and test samples were both internal transcribed spacer 1 (ITS1) DNA. We furthermore assume that the generation of sample ASVs and genera abundances was as accurate as that for the mock community such that the output sequencing data reflects relative abundance of the genera in the total population. Furthermore, by using the PowerSoil kit, we assumed that the DNA of all fungal species was efficiently extracted to represent the fungal community on kernels. This is coupled with the non-significant loss of viable fungal spores (<0.6% loss at 95% confidence level) due to washing during the pellet preparation (see Data Set S4 in the supplemental material). In addition to this, the robustness of the sequencing was tested on basis of *Aspergillus* by pairing HDSeq data with dilution plating data. It should be noted that despite the two being different methods of fungal enumeration, they are expected to produce a good level of positive correlation of abundances. The result showed a significantly strong positive correlation in *Aspergillus* between HDSeq abundance (%) and dilution plating quantities (CFU/g) (Spearman’s rank correlation rho = 0.78; *P* < 0.001). Abundances between plating and sequencing are provided in Data Set S6 in the supplemental material.

### Preharvest maize mycobiome structure.

**FIG 1:**
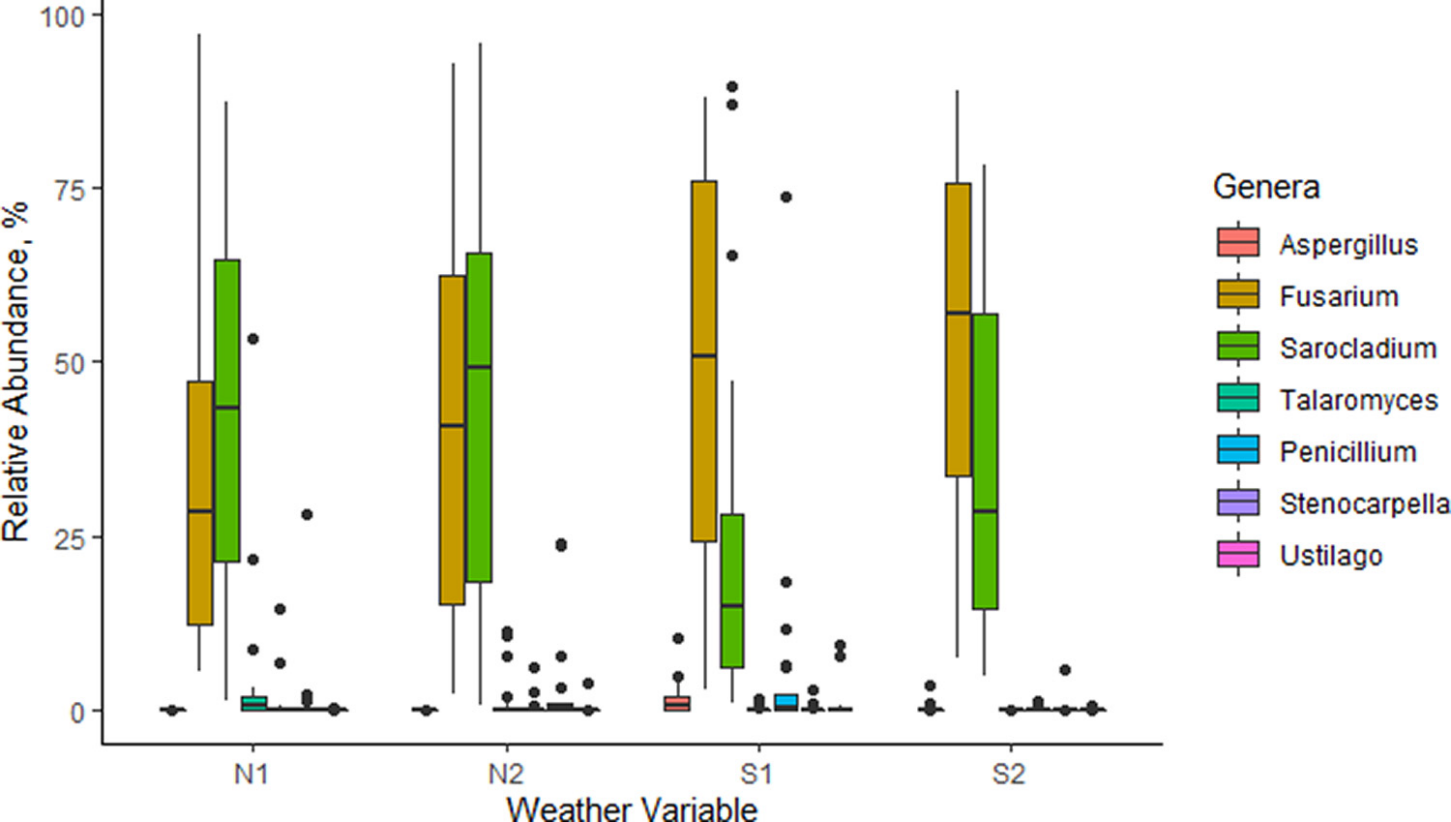
Boxplot of relative abundance representation of ASVs of the mycotoxin-important genera (*Aspergillus*, *Fusarium*, and *Penicillium*); the high ASV abundance *Sarocladium*; and three genera harboring common plant pathogens (*Stenocarpella*, *Talaromyces*, and *Ustilago*). N1 and N2 are high rainfall weather variables under AEZ3, season-1 and season-2, respectively. S1 is low rainfall with dry spell weather variable under AEZ1, season-1. S2 is low rainfall weather variable under AEZ1, season-2.

**FIG 2:**
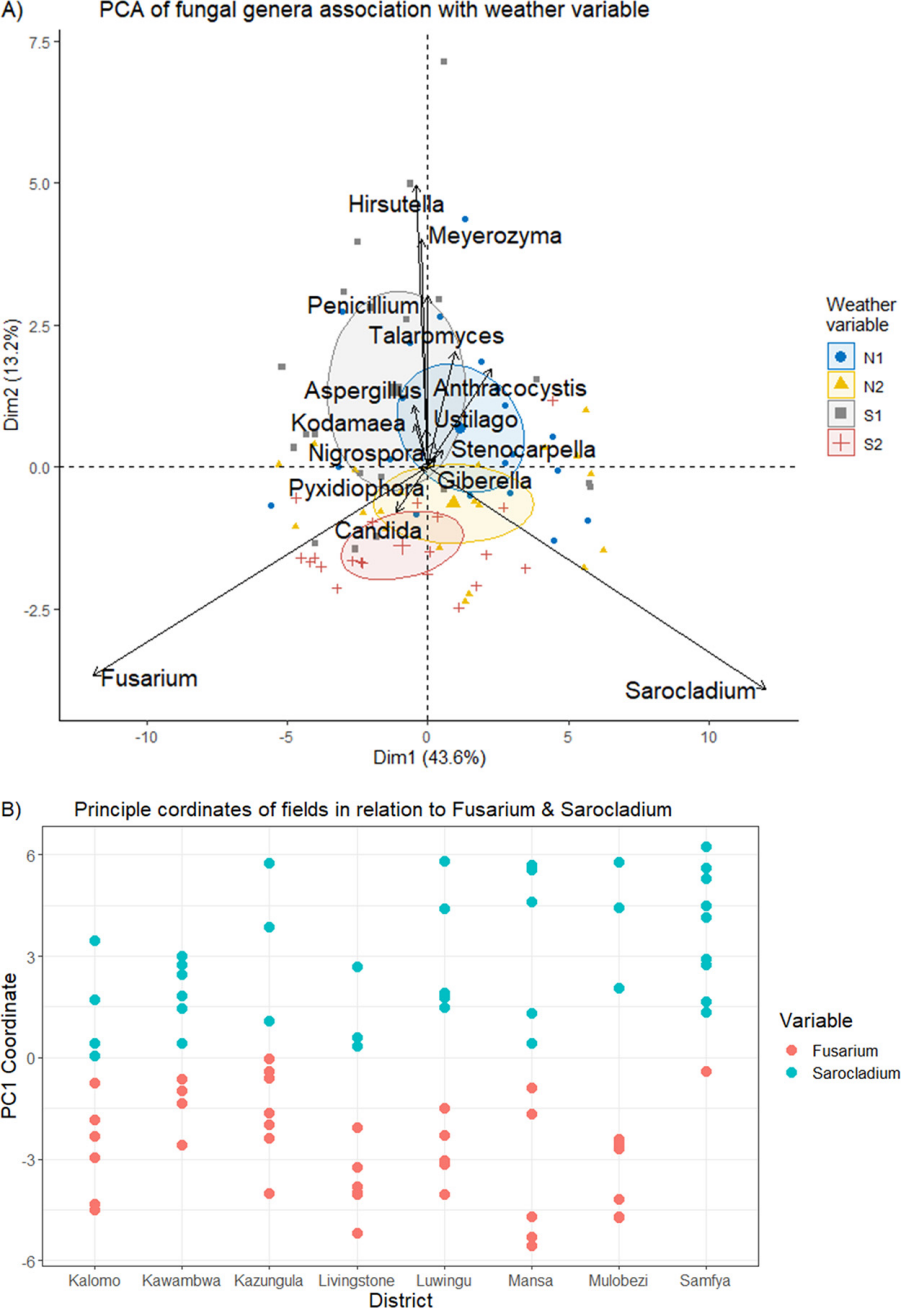
(A) Independent variables determining the inclination of the fungal genera. N1 and N2 are high rainfall weather variables under AEZ3, season-1 and season-2, respectively. S1 is low rainfall with dry spell weather variable under AEZ1, season-1. S2 is low rainfall weather variable under AEZ1, season-2. In the PCA biplot *Fusarium* (*equiseti*) is detected as a distinct anamorph of the former *Gibberella* (*intricans*). (B) Location-based (district) orientation of the principal-component analysis (PCA) coordinates for the two highest ASV abundance genera (*Fusarium* and *Sarocladium*) that had no weather variable orientation on the Biplot in panel a. Samfya district PCA coordinates for fields are biased in positive direction, as exceptionally dominated by *Sarocladium* (9/10 fields) than *Fusarium*.

Based on pooled data from agroecological zone 1 (AEZ1) and AEZ3 and mycobiome census, a total of 61 fungal genera were detected. *Sarocladium* was detected in high frequency (100%) throughout the fields. Furthermore, with respect to mycotoxin-important fungi, *Fusarium* was the dominant genus similarly with a 100% field frequency of detection like *Sarocladium*. In addition, *Fusarium* and *Sarocladium* had the highest ASV representation of 45% and 37%, respectively (Fig. 1). Although *Fusarium* and *Sarocladium* had the highest ASV representations across locations (districts), an exception was observed with Samfya district. The district had a lower average relative abundance of ASVs of *Fusarium* (17%) and a higher average for *Sarocladium* (60%). The exception is further demonstrated by the positions of the principle coordinates for *Sarocladium* under district Samfya, which are highly biased toward the arbitrary positive direction from the zero axis (Fig. 2B). For the rest of the districts, coordinates for *Fusarium* and *Sarocladium* evenly distributed across the zero axis toward the arbitrary positive and negative values. *Aspergillus*, a mycotoxin-important genus like *Fusarium*, had a 10 to 100% field detection frequency, which was weather variable dependent, highly detected under the low rainfall with dry spell variable S1 (Table 1). *Penicillium*, a reported common maize contaminant, had a high frequency of field contamination with average 76% across the weather variables. In combination with its former teleomorphic state, now separate genus *Talaromyces*, field frequency was 88%. We analyzed *Penicillium* and *Talaromyces* as separate genera considering their distinction in phylogenetic clades, with *Talaromyces* (member of Leotiomycetes [22]) having seven sections and *Penicillium* (member of Eurotiomycetes) having 26 sections (23). In Table 1, the common disease agent *Stenocarpella* as well as *Ustilago* were also frequently detected in fields with overall frequencies of 59% and 53%, respectively. Rare taxa, such as *Acremonium*, some of whose species are known human keratitis agents (24), were also reported. *Acremonium* had a low field frequency detection (6%) across the sampled locations (districts).

**TABLE 1:** Frequency and abundance of genera that were frequently detected including the mycotoxin and disease-burden important taxa

Genus	Weather variable	Frequency (%)	Relative abundance (%)	Mean relative abundance (%)	Relative abundance range (%)	
					Minimum	Maximum
*Fusarium*	S1	100	48.4	45	2.9	87.9
	N1	100	33.8		5.4	97.0
	S2	100	55.0		7.5	89.0
	N2	100	42.1		2.2	92.7
*Sarocladium*	S1	100	24.8	37	0.9	89.7
	N1	100	42.6		1.3	87.3
	S2	100	34.1		4.8	78.4
	N2	100	44.8		0.8	95.7
*Talaromyces* ^*a*^	S1	65	0,2	1.7	0,0	1,7
	N1	100	4,8		0,0	53,5
	S2	20	0,0		0,0	0,1
	N2	70	1,7		0,0	11,3
*Penicillium* ^*a*^	S1	95	6,0	2.0	0,0	73,7
	N1	80	1,2		0,0	14,7
	S2	65	0,1		0,0	1,3
	N2	65	0,5		0,0	6,3
*Stenocarpella*	S1	60	0.2	1.4	0.0	3.0
	N1	75	1.7		0.0	28.3
	S2	20	0.3		0.0	5.8
	N2	80	3.2		0.0	23.9
*Ustilago*	S1	90	1.0	0.3	0.0	9.3
	N1	45	0.0		0.0	0.3
	S2	65	0.0		0.0	0.7
	N2	10	0.2		0.0	3.9
*Aspergillus*	S1	100	1.7	0.5	0.0	10.3
	N1	40	0.0		0.0	0.1
	S2	40	0.3		0.0	3.4
	N2	10	0.0		0.0	0.0

a *Talaromyces* and *Penicillium* were analyzed separately considering the distinct phylogenetic clades, despite morphological resemblance. N1 and N2 are high rainfall weather variables under AEZ3, season-1 and season-2, respectively. S1 is low rainfall with dry spell weather variable under AEZ1, season-1. S2 is low rainfall weather variable under AEZ1, season-2.

### Mycobiome composition in relation to weather patterns.

**FIG 3:**
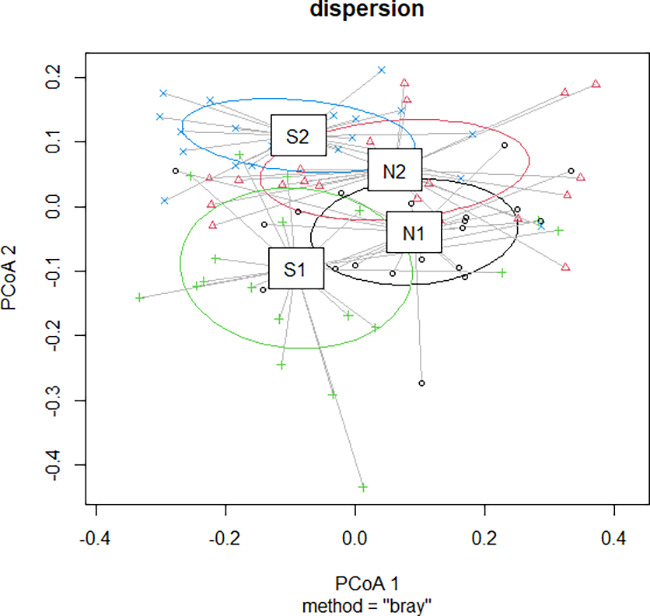
Principle Coordinate Analysis of fungal genera composition across weather variables. N1 and N2 are high rainfall weather variables under AEZ3, season-1 and season-2, respectively. S1 is low rainfall with dry spell weather variable under AEZ1, season-1. S2 is low rainfall weather variable under AEZ1, season-2. Difference in composition between S1 and S2 by pairwise adonis2 PERMANOVA test was significant, *P = *0.006. N1 versus N2, not significant.

**FIG 4:**
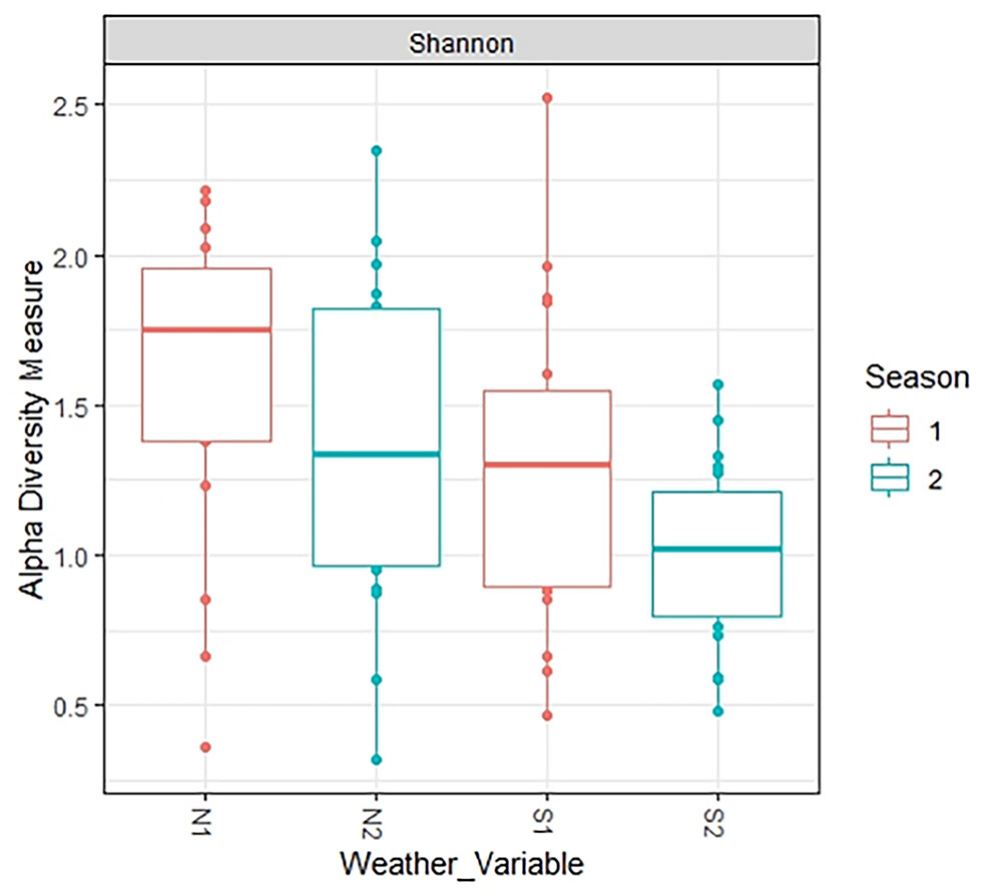
Alpha diversity measured by precision of detected species in mycobiomes of the weather variables. N1 and N2 are high rainfall weather variables under AEZ3, season-1 and season-2, respectively. S1 is low rainfall with dry spell weather variable under AEZ1, season-1. S2 is low rainfall weather variable under AEZ1, season-2.

Aspects of mycobiome composition in relation to weather variables S1 (low rainfall with dry spell), N1 (high rainfall), S2 (low rainfall), and N2 (high rainfall) are shown in Fig. 2, 3, 4, and 5 as well as in Table 2. A difference in fungal composition was detected between S1 (low rainfall with dry spell) and S2 (low rainfall) (Fig. 3) (*P = *0.006). However, there was no difference in fungal composition between the high rainfall variables N1 and N2 (*P = *0.492). A comparison of weather variables, N1, N2, S1, and S2, within an AEZ showed that there was no difference in alpha diversity between N1 and N2 as well as between S1 and S2. However, a significant difference in alpha diversity was observed between N1 and S2 (Fig. 4) (pairwise Tukey’s honestly significant difference [HSD], *P = *0.03).

**FIG 5:**
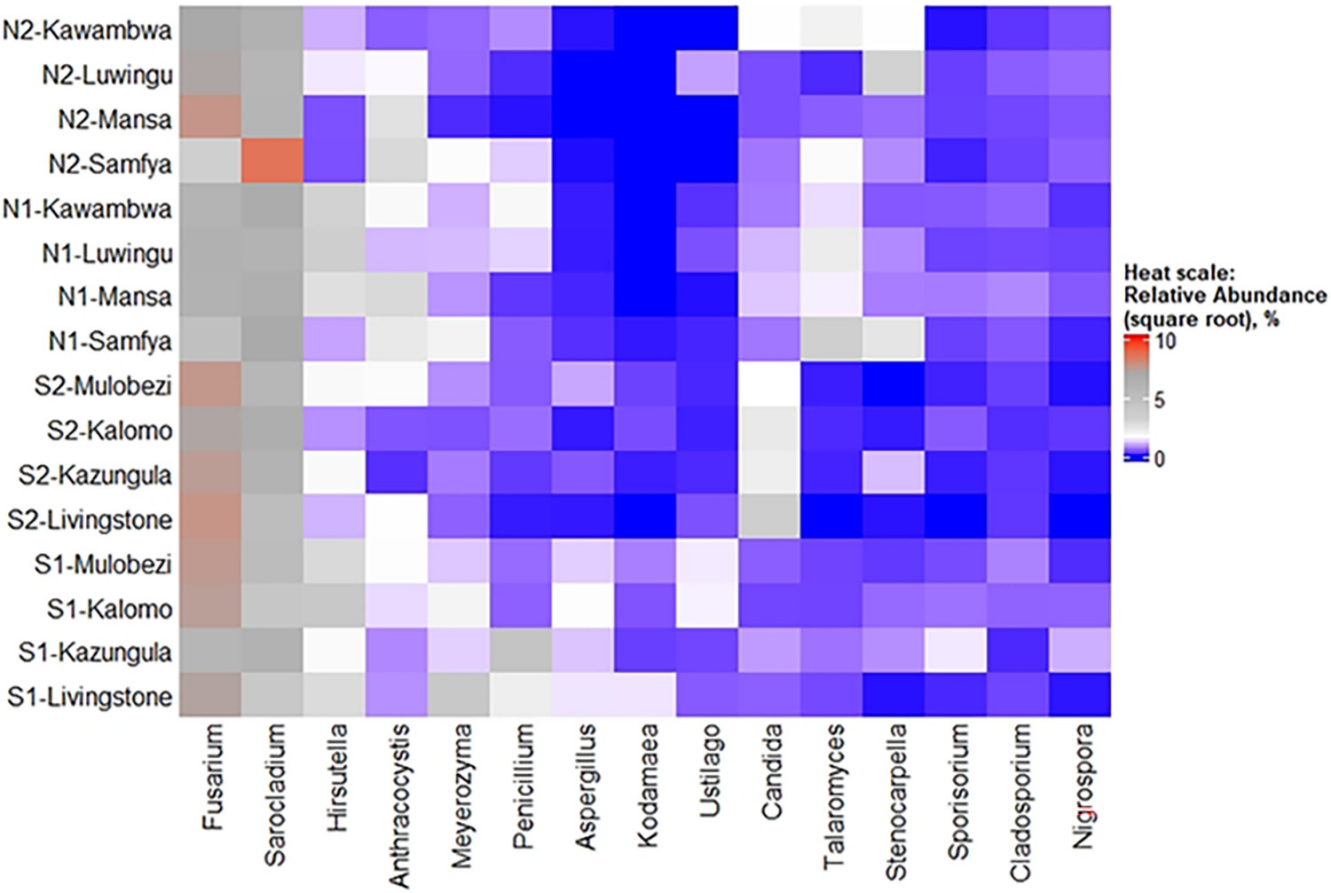
Unclustered heatmap of top 15 fungal genera (*x* axis) relative abundances across districts over two seasons (*y* axis). N1 and N2 are high rainfall weather variables under AEZ3, season-1 and season-2, respectively. Suffixes are districts. S1 is low rainfall with dry spell weather variable under AEZ1, season-1. S2 is low rainfall weather variable under AEZ1, season-2.

**TABLE 2:** Mean relative abundance describing the distinct fungal genera over weather variables^*a*^

Genus^*b*^	Mean relative abundance			
	S1	S2	N1	N2
Aspergillus*	1.7	0.3	0.0	0.0
Kodamaea*	0.6	0.1	0.0	0.0
*Meyerozyma*	5.4	0.3	1.6	0.8
Penicillium*	6.0	0.1	1.2	0.5
*Talaromyces*	0.2	0.0	4.8	1.7
*Stenocarpella*	0.2	0.3	1.7	3.2
*Ustilago*	1.0	0.0	0.0	0.2
*Hirsutella*	8.7	1.9	7.2	0.8
Fusarium taxon^*c*^	0.2	0.1	2.0	2.7

a Table 2 shows changes in relative abundance of fungi over the different weather variables.

b Genera marked with an asterisk (*) are xerophilic/moderately xerophilic, while the rest have some agroecological zone inclination.

c Detected as *Fusarium equiseti*, a distinct anamorph of the former Gibberella intricans.

Between AEZs, combined data showed that the drier AEZ1 had a comparatively higher *Fusarium* relative abundance (52%) than AEZ3 (38%) (Student's *t* test, *P = *0.028) (Fig. 5). However, there was no difference in relative abundances of *Fusarium* over the weather variables, S1 (48.4%), S2 (55.0%), N1 (33.8%), and N2 (42.1%) (pairwise *t* test, *P* > 0.05). Similarly, *Aspergillus*, an important mycotoxins genus like *Fusarium*, was substantially more relatively abundant in AEZ1 than in AEZ3 (Kruskal-Wallis rank sum test, *P* < 0.001, χ^2^ = 22.71, df = 1). A comparison of weather variables showed that *Aspergillus* abundance was higher under the S1 compared to all three variables N1, S2, and N2 (pairwise Wilcoxon rank sum test, *P* < 0.001). The differences between AEZ1 and AEZ3 are further elaborated in Fig. 2A as follows: dimension 1 (Dim-1) explains more the difference in mycobiome variation in AEZ1 and AEZ3. Dim-2 explains more the difference between season-1 and season-2, indicating *Hirsutella* to be more associated with season-1 than season-2. Overall, Dim-1 explains variations better (43.6%) than Dim-2 (13.2%). It is evident from Fig. 2A that *Penicillium*, *Kodamaea*, *Aspergillus*, and *Meyerozyma* were more associated with S1, whereas *Fusarium* and *Sarocladium* are not associated with a particular weather variable.

Within the AEZ1, some genera had significantly higher relative abundance (*P* < 0.01) in the first season characterized by a dry spell (weather variable S1) compared to the following sampling season (weather variable S2) (Table 2). In particular, *Aspergillus* relative abundance was higher under S1 than S2. Furthermore, the more xerophilic members (*) were more associated with S1. Meyerozyma was more associated with season-1 than 2 and proliferated the most under S1, while the rest of genera were either associated with AEZ3 or AEZ1. No such significant differences were observed between the two high rainfall variables, N1 and N2.

### Correlation between genus level *Aspergillus*/*Fusarium* and aflatoxin/fumonisin-B1.

The pattern of occurrence of aflatoxin (AF) and fumonisin-B1 (FB1) corresponded with the pattern of relative abundance of *Aspergillus* and *Fusarium*, respectively.

Field AF contamination resonated with genus *Aspergillus* contamination (Spearman's rank correlation rho = 0.78, *P < *0.001) as well as *Aspergillus* section *Flavi* quantities (CFU/g) (Spearman's rank correlation rho = 0.64, *P < *0.001). The AF contamination was only detected in maize under weather variable S1 (low rainfall with dry spell) (Table 3). Although the AF contamination geometric mean for S1 was low (6.7 μg/kg), levels in individual samples ranged from nondetectable to 325 μg/kg, with at least one field in each district (Kalomo, Kazungula, Livingstone, and Mulobezi) showing total AF levels higher than 50 μg/kg. In addition, 10/20 fields had AF exceeding the EU limit of 4 μg/kg, with 9/20 exceeding the southern and eastern African regional trading block (COMESA) limit of 10 μg/kg. No field AF was detected in the same region (AEZ1) when the rainfall pattern was the normal low rainfall (S2). Equally, no AF was detected in maize samples from high rainfall zone AEZ3 in both seasons (N1 and N2).

**TABLE 3:** Frequency of genus *Aspergillus* in fields and level of aflatoxin contamination

Weather variable	No. of fields positive for *Aspergillus* on maize/total no.	No. of fields positive for aflatoxin on maize/total no.	Aflatoxin (total) (μg/kg)^*a*^
S1	20/20	10/20	ND–352
N1	8/20	0	ND
S2	8/20	0	ND
N2	2/20	0	ND

a ND, not detected.

Similarly, FB1 contamination followed the pattern of *Fusarium* occurrence (relative abundance) although the correlation was weaker (Spearman's rank correlation rho = 0.31, *P = *0.005) compared to that for AF and *Aspergillus*. AEZ1 recorded higher levels of FB1 in maize (geometric mean 83.9 mg/kg) than AEZ3 (geometric mean, 14.2 mg/kg; *t* test log-FB1, *P = *0.002). Among the studied weather variables, FB1 contamination was highest in S1 and lowest in N2, the latter of which had significantly lower FB1 levels compared to those of the other weather variables. Data Set S8A and B in the supplemental material shows details of the AF and FB1 contamination in samples across sampled locations and weather variables.

## DISCUSSION

Deploying high-throughput DNA sequencing (HDSeq) of the ITS1 region, we describe and show a diverse fungal microbiome on preharvest Zambian maize in comparison to previous findings. In addition, we demonstrate the dominance of the previously unreported *Sarocladium* on Zambia preharvest maize, which is also seldom reported in other regions. We furthermore demonstrate for the first time how rainfall pattern may impact the composition of a diverse mycobiome.

### Preharvest maize mycobiome structure.

Although the previous study done in Zambia by dilution plating revealed 13 genera on the maize at harvest (5), we reveal a 5-fold higher number of genera (*n *= 61) (excluding genera that only appeared in one field with a relative abundance of <0.1%; see Data Set S2 in the supplemental material). This demonstrates a higher diversity of the preharvest maize mycobiome than previously reported. We attribute the higher number of preharvest fungal genera detected to the use of a higher resolution method (HDSeq) compared to dilution plating. Standard techniques for quantifying fungal populations, such as dilution plating, although quite effective, may tend to favor quiescent structures, particularly conidia, compared to other actively growing parts of the population (25). Furthermore, although effective in fungal enumeration, dilution plating may favor the most abundant species over the less abundant and slower growers, which may result in the underrepresentation of the latter (26–28). Hence, using HDSeq would likely give a more realistic picture of fungal diversity than plating methods (28–31). Previous studies (26, 27) similarly demonstrated a higher number of detected genera by HDSeq compared to plating. Although we do observe a common pattern of the common maize contaminants *Aspergillus*, *Fusarium*, *Penicillium*, and *Stenocarpella* from our study and the previous study on U.S. and Kenyan maize using HDSeq (26), our study does report unique genera in high frequency. These include the maize pathogen *Ustilago* (8) (53% frequency), *Kodamaea* (31% frequency), *Meyerozyma* (88% frequency), and *Anthracocystis* (94% frequency).

Out of 61 genera detected on Zambian preharvest maize, *Fusarium* and *Sarocladium* were the most frequently detected genera across all fields (100%) (Table 1). Furthermore, we demonstrate that although *Sarocladium* has previously not been reported on Zambian maize, it was a highly frequent contaminant of maize in the fields like *Fusarium*. Our findings contrast expectations of a preharvest mycobiome dominated by *Fusarium* and *Stenocarpella*. A past dilution plating study of Zambian maize collected from AEZ1 and AEZ2 demonstrated *Fusarium* to be the fungus of highest abundance on the preharvest maize out of the 13 genera that were detected (5). However, *Sarocladium* was not detected. *Sarocladium* is also rarely reported in regional studies of similar climatic pattern as Zambia but may be a fungus of importance in contaminating preharvest maize. For example, a recent regional study by plating similarly revealed *Fusarium* as the main contaminant of maize in Zimbabwe (32), but *Sarocladium* was not reported. A different climatic region (26) reported similar high relative abundances of operational taxonomic units (OTUs) for *Sarocladium* in Kenyan and U.S. maize using HDSeq. Although *Sarocladium* may be important to the maize mycobiome according to our study, it should also be noted that overall, the genus is largely a rice pathogen (33). Compared to *Fusarium*, *Sarocladium* has no such reported level of pathogenicity or mycotoxigenicity on maize. Regarding *Fusarium*, our findings for AEZ3, as well as the previously studied AEZ1 in reference 5 and this study, confirm that *Fusarium* is readily detected on preharvest Zambian maize. The dominance of *Fusarium* by frequency of detection is also demonstrated by Lane et al. (26) on Kenyan and U.S. maize. This further demonstrates that *Fusarium* is a widely important field maize contaminant considering that, in combination, our study and the previous study (26) were done under three different geographical regions. Although the actual abundance of *Fusarium* may not have been ascertained in this study, we reveal a similar pattern of the comparatively high abundance of *Fusarium* by ASVs (45%) as that of previous study of *Fusarium* OTUs using HDSeq (26).

Although our study shows that *Fusarium* was a genus present at high frequency in the field throughout weather variables, better climatic associations would be clearer if the genus were split into various species based on ecological adaptations. For example, accounting for about 3% of *Fusarium* in this study, *Fusarium equiseti* (genetically distinct in this study as the former Gibberella intricans [34]), was more associated with districts in AEZ3 (2.35%) than AEZ1 (0.17%). However, it is unclear from this study if the assigned taxon *Fusarium* were split further into species, that certain *Fusarium* species would be seen to incline toward a particular AEZ or weather variable studied. Furthermore, a similar association of genus with specific weather variable is observed with *Candida*, which was more associated with the southern hotter and drier region (AEZ1) under a normal rainfall pattern (S2) (Fig. 2A). We also see a pattern in which, over the two maize growing seasons, the biological state *Talaromyces* (64% frequency) was found to be more commonly associated with the areas of AEZ3 than in AEZ1 similar with *Stenocarpella* (Fig. 5 and Fig. 2A). It should be noted that *Talaromyces* has a distinct phylogenetic clade (with seven sections) from its asexual anamorph *Penicillium* (with 26 sections) (23) and is, hence, assessed separately from *Penicillium* in our study. *Talaromyces* is a genus recently reported as causative in destructive maize ear rot (35). An earlier country study has demonstrated *Fusarium* and *Stenocarpella* to be causative fungi of maize ear rot in Zambia (5). The higher relative abundance of *Talaromyces* observed in the wetter northerly districts (AEZ3) compared to the drier southerly districts (AEZ1) may augment the maize ear rot caused by *Fusarium* and *Stenocarpella*. This warrants the need to further explore species within *Talaromyces* and establish further links between maize ear rot and this genus in AEZ3. It should also be noted that some species belonging to this genus are known for the production of the mycotoxin rubratoxin (36) like some *Penicillium* species (37).

Of the mycotoxin-important genera other than *Fusarium*, *Aspergillus* was part of the most frequently detected genera in fields investigated (Table 1). A previous study (5), which partly investigated districts in AEZ1 showed *Aspergillus* abundance to be the second highest after *Fusarium*. In our study, however, we detected an additional four genera between *Fusarium* and *Aspergillus* in order of reducing frequency of appearance in AEZ1, namely, *Sarocladium* (100%) > *Hirsutella* (95%) > *Penicillium* (80%) > *Ustilago* (78%), with *Penicillium* being important for the mycotoxin ochratoxin. *Aspergillus* frequency in AEZ1 was 70%, indicating its importance in this AEZ. It is also worth noting that although *Aspergillus* was in comparatively lower relative abundance in the field for the low rainfall variable S2 compared to the low rainfall with dry spell variable S1 (Tables 1 and 2), propagules of this moderately xerophilic genus carried over from field to storage can proliferate during storage (38) especially when conditions become favorable.

### Mycobiome composition in relation to weather patterns.

We demonstrate that although generally stable, the preharvest mycobiome composition may be altered by such conditions as a dry spell. The stability of the mycobiome composition in our study is demonstrated in Fig. 3. It is further exemplified by the nonsignificant difference in the relative abundance of the high ASV abundance *Fusarium* over weather variables S1 (48.4%), S2 (55.0%), N1 (33.8%), and N2 (42.1%) (pairwise *t* test, *P* > 0.05) (Table 1). The stability in mycobiome composition is despite specific inclinations of certain genera like *Talaromyces* and *Stenocarpella*, toward AEZ3 and *Candida* toward S2 (Fig. 5). As regards the change in composition, the low rainfall variable with dry spell (S1) had a demonstrably different composition from that of the normal low rainfall variable (S2). The difference in composition between S1 and S2 is attributed to the proliferation of the moderately xerophilic members *Aspergillus* and *Penicillium* as well as the less-reported *Kodamaea* in S1, similarly with *Meyerozyma*. When rainfall normalized in the same locality the following sampling season (season-2, 2020/2021), the genera relative abundances declined (Table 2). The three genera, as xerophiles, as well as *Meyerozyma*, would be more adapted to the dry spell conditions (S1). Considering that certain genera like *Kodamaea* appeared under the dry weather pattern, it may be imperative for future studies to consider the dynamics of the relative abundance of such species during storage. In addition, consideration should be taken to explore if such genera are specific to certain geographic regions at all or are truly weather pattern dependent (S1) as seen in our study.

**FIG 6:**
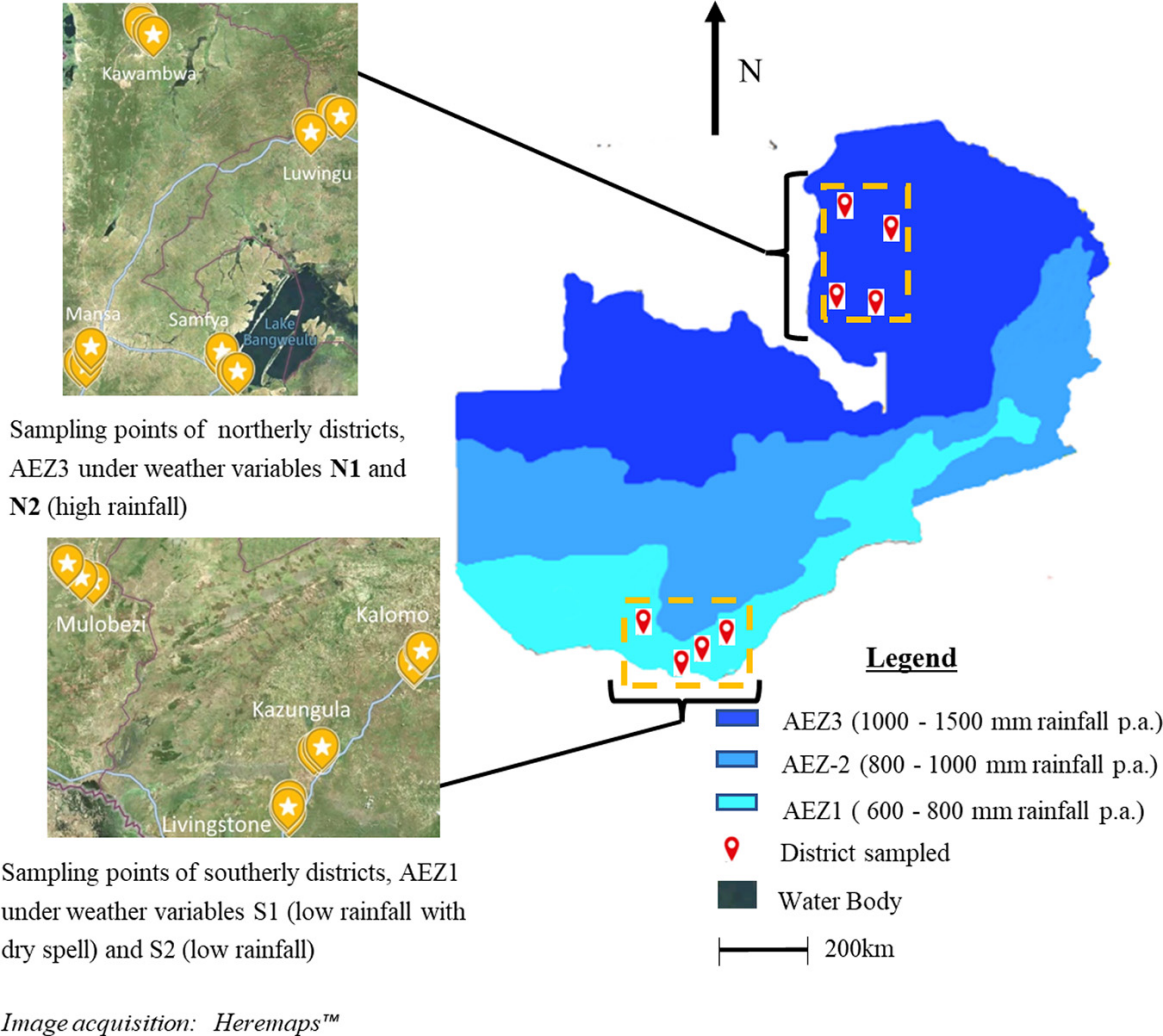
Zambia’s agroecological zones and sampling points from selected districts.

Regarding the mycotoxin-important genera *Aspergillus* and *Fusarium*, the strong influence of the dry spell was the key factor that led to *Aspergillus* proliferation (Fig. 2A) as has been shown in earlier studies (39, 40). *Aspergillus* contamination observed under S2 and its strong proliferation under S1 demonstrates the potential risk of field contamination of maize with *Aspergillus*, a fungus generally associated with the maize storage phase. On the contrary, this study revealed that *Fusarium* abundance on the preharvest maize was not influenced by the dry spell conditions. This may imply that maize ear colonization by *Fusarium* may likely have occurred early before onset of the dry spell (about 3 months after sowing). An exception was, however, observed in the Samfya district (under high rainfall zone, AEZ3), where the comparatively lowest relative abundance of *Fusarium* and inversely the highest relative abundance of *Sarocladium* was observed. This may be attributed to the fact that Samfya’s agroecological terrain is, in part, comprised of lakes as water bodies (Fig. 6) conducive for the better adapted rice pathogen *Sarocladium* (41, 42). This also suggests that, although our study and a previous study (26) showed *Fusarium* to have the most abundant ASVs/OTUs on maize, *Fusarium* may probably not always be the most abundant taxon on maize in comparison to *Sarocladium*. This argument applies to regions such as rice growing areas with abundant water bodies, such that *Sarocladium* would instead dominate. For example, a study (43) recorded incidences of *Sarocladium* infection of maize without concurrent infection by *Fusarium*. Considering that *Sarocladium* and *Fusarium* were found to be the main contaminants of preharvest maize, it is worth exploring whether or not certain species of *Sarocladium* could have a bearing on levels of fumonisin (FB) in preharvest maize based on their relative abundances.

### Correlation between genus level *Aspergillus*/*Fusarium* and aflatoxin/fumonisin-B1.

Although there was a strong correlation between levels of AF and genus *Aspergillus* (as well as *Aspergillus* section *Flavi*), it could not be ascertained which species of *Aspergillus* were responsible for the AF contamination of the maize, as taxa were resolved up to the genus level. However, considering that AF contamination was strongly correlated with *Aspergillus* proliferation, this may demonstrate that AF-producing species are likely to be commonly present whenever the genus is detected. Some studies, for example, have demonstrated scenarios where nearly all *Aspergillus* section *Flavi* species detected were toxigenic (44). In our scenario, this would hence lead to the field AF manifestation as seen under severely dry weather pattern such as S1. Although AF is essentially considered a storage mycotoxin in maize, our results consolidate the fact that the maize in the field is not void of high AF contamination, supported by other findings (40, 45).

Similarly, the relative abundance of *Fusarium* correlated with levels of FB1 in maize. However, we could also not establish which *Fusarium* species were responsible for the FB1 contamination in the maize, as *Fusarium* was resolved up to genus level. However, the correlation between *Fusarium* abundance and FB1 signifies the importance of *Fusarium* at genus level in the contamination of the maize with FB1. This demonstrates that FB1 is an important part of the *Fusarium* mycotoxins. It furthermore suggests that notorious species of *Fusarium* producing FB1 are likely to be a common integral part of the genus *Fusarium*. This is despite the fact that Aspergillus niger is also an FB producer (46) although not a part of the genus *Fusarium*. Aspergillus niger would, however, not have been expected to have a major influence on FB levels given that levels of the genus *Aspergillus* were in much lower abundance compared to *Fusarium*, particularly under N1, N2, and S2. Considering that the contamination of *Fusarium* was perpetual irrespective of weather variable, this does show the risk of field maize contamination with FB1 and may in addition explain the constant contamination of field maize in Zambia with FBs (47). We do demonstrate in this study that FB1 contamination appeared to be associated with AEZ unlike AF, which was weather variable dependent. The districts of AEZ1 had significantly higher levels of FB1 than districts of AEZ3. The FB1 pattern resonates with a long-term study done on field maize in Serbia, which showed higher levels of FB being associated with drier climatic conditions (48), characteristic of AEZ1 in our study.

### Conclusion.

The mycobiome of preharvest Zambian maize is more diverse than previously reported. Furthermore, the previously unreported *Sarocladium* may be a key contaminant of preharvest maize besides commonly reported *Fusarium*. Although the fungal composition and diversity were similar over the different rainfall patterns, severe weather conditions, such as dry spell, may alter the mycobiome composition due to the proliferation of more xerophilic genera supported by such conditions. The high prevalence of *Sarocladium* and *Fusarium* warrants the need to further study the species of *Sarocladium*, as it could be a fungus of interest from a phytosanitary perspective with respect to *Fusarium* and FBs in maize. It's hereby noted that although not all species of *Aspergillus*/*Fusarium* are AF/FB1 producers, the genus level of *Aspergillus*/*Fusarium* resolution was able to provide a good level of prediction for preharvest maize AF/FB1 contamination.

## MATERIALS AND METHODS

### Study area and assignment of weather variables.

Two regions of Zambia with known contrasting climatic patterns, dry and hot versus wet and comparatively cooler, were selected for the study. The first region was to the south (S) of Zambia (within latitude 16° 36' 54” S to 17° 46' 40” S and longitude 24° 49' 57” E to 26° 31' 35” E), from which four districts were selected for the study (Fig. 6), and is part of Zambia’s agroecological zone 1 (AEZ1). The second region was in the north (N) of Zambia (within latitude 9° 44' 55” S to 11° 30' 21” S and longitude 28°46' 35” E to 30° 5′ 40” E), from which four districts were selected for the study, and is part of AEZ3. The two AEZs are climatically contrasting regions such that on average AEZ1, characterized as a low rainfall area, receives less rainfall (600 to 800 mm) per annum (p.a.) and is hotter (30 to 36°C) than AEZ3. AEZ3, characterized as a high rainfall zone, receives higher rainfall (1,000 to 1,500 mm) p.a. and has comparatively lower average temperature (30 to 33°C) (21, 49) (https://www.yieldgap.org/zambia; live running year meteorological data available at Zambian online weather portal, http://41.72.104.142:8080/livedata/map.jsf). For example, between 2010 and 2012 from this historic data, Mansa district (under AEZ3 in our study) received 1,367 (±189) mm rainfall with mean maximum annual temperature of 32.5°C (±0.4°C). In the same period, Livingstone district (under AEZ1 in our study) received 695 (±137) mm rainfall with mean maximum annual temperature of 36.3°C (±0.5°C). The collection of preharvest field maize was carried out over two maize cropping seasons in 2018/2019 (season-1) and 2020/2021 (season-2). For this study, AEZ with interaction of maize cropping season was defined as weather variable “S1” (AEZ1 in season-1), “N1” (AEZ3 in season-1), “S2” (AEZ1 in season-2), and “N2” (AEZ3 in season-2). The variables were defined by rainfall pattern as low rainfall with dry spell (S1), low rainfall (S2), and high rainfall (N1 and N2). During the study, S1 had a prolonged dry spell during the maize growth season-1 from 15th February to early April 2019, followed by about one to three precipitations for about a week. S2 had stable rains according to normal conditions of AEZ1. Similarly, N1 and N2 had stable rains according to average weather conditions of AEZ3.

### Sampling.

Sampling per AEZ was carried out immediately after farmers began harvesting, mid-April for AEZ1 (low rainfall zone) and early June for AEZ3 (high rainfall zone). The timing of the sample collection was done with prior consultation with field agricultural extension officers. Per sampling period, four districts were selected from each AEZ. Selection of districts was on the basis that they were of agricultural importance by production tonnage of maize, human population, and accessibility to commerce in terms of sales of harvest to commercial crop marketing agencies/enterprises. Per district, five maize fields were purposively selected for sampling. Therefore, 20 fields were sampled in each AEZ per sampling period. Consequently, a total of 80 maize fields were sampled for the entire study.

In both study regions, maize field sizes varied between 1.0 and 2.5 acres. A minimum of 1 km between selected sampling fields was made to avoid possible influence of neighboring fields’ mycobiomes on each other. The collection of maize cobs per selected field was executed according to the method described previously (50). A total of 30 maize cobs per field were collected along two diagonal transects. For season-1, cobs with poor husk cover were substituted with the nearest well covered cobs to avoid chances of superficial fungal contamination from the soil or air. For season-2 sampling, an equal number of good husk covered and poor husk covered cobs (*n* = 15 per set) per field were collected. For all collections, the maize cobs were retained in their husks and placed in permeable potato bags and weighed. On-site moisture determination (see Data Set S1 in the supplemental material) was carried out using a portable moisture meter (model MT-Pro+, AgraTroinx, OH, USA). This was done on two extra cobs collected randomly from two separate points in the field over the two diagonals.

### Sample preparation.

Samples were dried without removing husks (42 ± 2°C, 48 h) to a moisture content of about 8 to 13% in a forced draft oven (model D-6450; Heraeus, Hanau, Germany). This was done to prevent any potential growth of fungi or mycotoxin build up. The dried samples were then shelled, without touching the kernels, with a sterilized clean rod in a sterile polyvinyl chloride (PVC) bag. For both seasons, the shelled grains from one transect with good-husk cover cobs were thoroughly homogenized and split into two approximately equal parts and stored at −35°C in freezer (model 3565 S; Scientemp, MI, USA) pending further analyses. One portion was for mycotoxins analysis while the other part was for mycobiome assays.

Prior to milling, the entire grain subsample for mycotoxins analysis was further homogenized in a 10-kg tumble blender (model MB015; Pharmatech, Warwickshire, UK) for 10 min at 20 rpm. About 1 kg homogenized grain sample was milled using an Ultra Centrifugal Mill (model ZM200; Retsch, Haan, Germany) fitted with a 1.0-mm ring sieve. All material passed through the sieve during milling. Prior to mycotoxin analysis, the milled material was further homogenized using a 1-kg tumble blender (model MB005; Pharmatech, Warwickshire, UK).

### DNA extraction.

All of the water used in the assays was Milli-Q type-1 water from a deionizer (Integral-3 Milli-Q water deionizer; Merck, Darmstadt, Germany) and was sterilized by autoclaving at 120°C for 15 min. Prior to DNA extraction, a pellet from the maize surface wash containing target fungal DNA was generated. Like in a similar study, we used centrifugation to pelletize the wash (26).

In our protocol, for each sample 100 g, kernels were weighed into a sterile 250-mL Erlenmeyer flask. Next, 35 mL of sterile 0.05% Triton X solution was added to the flask. The flasks were shaken for 3 min at amplitude 12 on a shaker (Burrell Wrist Action shaker, model 75; Burrell Scientific, LLC, Pittsburgh, PA, USA). The contents were then swirled by hand for about 10 s to homogenize, and the solution was passed through a sterilized 1.2-mm nylon mesh into a sterile 50-mL polypropylene tube to separate kernels from the wash. The tube was centrifuged at 12,751 × *g* for 10 min in a temperature-controlled centrifuge (Suppra 22K; Hanil Scientific Inc., Gimpo, South Korea) set at 10°C. All of the 1st supernatant was removed, leaving about 3 mL with the pellet that had formed at the bottom of the tube. The pellet was resuspended by briefly vortexing, and two equal portions of the suspension were transferred to two 2-mL microcentrifuge tubes. The microcentrifuge tubes were centrifuged at 14,000 rpm for 5 min in a temperature-controlled centrifuge (model 5403; Eppendorf, Hamburg, Germany) set at 4°C. The 2nd supernatant was removed without agitating the pellet. To wash off the residue surfactant (Triton X), 1,000 μL of sterile water was added to the microcentrifuge tubes. The tubes were then vortexed to resuspend the pellet and recentrifuged at 4°C as in the previous step. The 3rd supernatant was removed, the washing step repeated, and the final 4th supernatant was separated from the pellet. The possible loss in spore quantity due to the multiple pellet washing was estimated by dilution plating on peptone-dextrose agar (PDA) (see Data Sets S3 and S4 in the supplemental material).

DNA, which included target fungal DNA, was extracted from the pellet using the PowerSoil DNA isolation kit (MO BIO Laboratories, Carlsbad, CA, USA). Four hundred microliters of CD1 lysis buffer was added to each of the two tubes containing sample pellet. The contents were vortexed, and the pellet suspensions from both tubes were transferred to one bead-beating tube. The prescribed PowerSoil DNA isolation kit protocol (https://qiagen.com/nl; kit catalog no. 47014) was used to isolate the DNA. Tube beating was carried out using a homogenizer (model MM200; Retch, Haan, Germany) set at 25 beats/s for 12 min continuously. The bead-beating tube was then centrifuged at room temperature in a microcentrifuge unit (model 5403; Eppendorf, Hamburg, Germany). The isolated DNA concentration was read on a spectrometer (Nanodrop model 2000; Thermo Scientific, Wilmington, DE, USA).

To test the robustness of the amplicon sequencing, a mock community of fungal DNA was generated and treated as samples alongside the season-2 samples. This was for quality control purposes to determine accuracy of the DNA recovery from PCR amplification, sequencing, raw data pipeline clean up, and finally the generation of amplicon sequence variants (ASVs) and fungal genera abundance. The mock community consisted of a pure Fusarium verticillioides isolate DNA representing high ASV relative abundance genus on maize and pure Aspergillus flavus isolate DNA representing the lower ASV relative abundance genus on maize. The DNA for spp. *verticillioides* and spp. *flavus*, upon dilution of each to 10 ng/μL, were mixed in the following percentage ratios in duplicate: 95:05, 85:15, 50:50.

**FIG 7:**
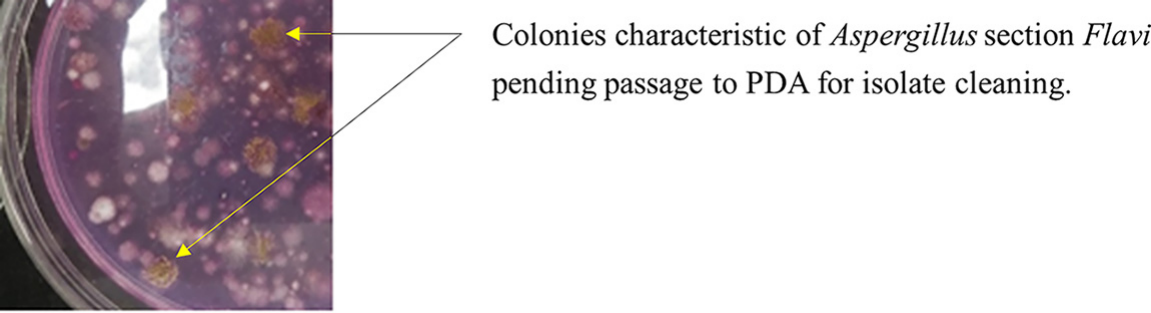
Isolation and enumeration of *Aspergillus* section *Flavi* from maize kernel wash on modified rose Bengal agar medium (MRB).

Furthermore, we cultured *Aspergillus* from maize kernels by dilution plating to compare fungal abundance by culture-based analysis with DNA amplicon sequencing. In this approach, *Aspergillus* section *Flavi* was quantified from kernels washed on modified rose Bengal agar (MRB) by dilution plating as previously described (51). Briefly, 40 g of kernels were transferred to a 250-mL sterile bottle. Next, 40 mL 0.05% sterile Triton X was added. Contents were shaken on a sideways shaker (GFL model 3018; Society for Laboratory Technology, Burgwedel, Germany) at 200 rpm for 10 min. This made a 1× initial extract. For the plating, 1 mL of the initial extract was diluted with sterile Milli-Q water to a 0.5× solution and then serially diluted up to 1 × 10^−3^, with agitation during each pipetting. Next, a 150-μL suspension was plated on 90-mm petri dishes of MRB and spread with the help of sterile 3-mm glass beads (20 to 30 beads per plate). MRB plates were incubated at 31°C (3 days, dark). Colonies were enumerated as CFU/gram kernels based on their characteristic morphology for *Flavi* (Fig. 7) (51). The culturing, in order to compare *Aspergillus* abundance by dilution plating with DNA amplicon sequencing, was also done on all of the 80 sample collections representing the different weather variables (S1, low rainfall with dry spell; S2, low rainfall; N1, high rainfall; N2, high rainfall).

### High-throughput DNA sequencing and bioinformatic analysis.

The purified template DNA concentration per sample was normalized to 10 ng/μL from initial concentrations determined on the Nanodrop spectrometer. The DNA was sequenced on the platform Illumina, Miseq V3, by paired-end amplicon sequencing (2 × 300 bp) at LGC Genomics (Biosearch Technologies, Berlin, Germany). The targeted region for the bidirectional sequencing was the ITS1 (nuclear ribosomal internal transcribed spacer 1) region of the fungal genomic DNA. Amplification of the ITS1 region, partly overlapping into the 5.8S region, was achieved using the following primer set: ITS1F_Kyo2 (forward) TAGAGGAAGTAAAAGTCGTAA and ITS86R (reverse) TTCAAAGATTCGATGATTCAC. We selected ITS1 in preference to ITS2 for its higher specificity for fungal DNA against non-fungal DNA (52). This is considering that environmental DNA samples would be highly laden with nontarget plant DNA.

The adaptor- and primer-clipped raw data after the sequencing was in the form of demultiplexed samples with an average sequencing depth of 131,351 (±26,239) total reads per sample for season-1 and 229,027 (±31,270) total reads per sample for season-2. All reads of a final length of <100 bp had been discarded. The raw data was then processed in the bioinformatics pipeline DADA2 (Divisive Amplicon Denoising Algorithm version 2) (53). This was for raw reads quality adjustment, assembly of forward and reverse reads, generation of ASVs from the merged reads, and the assigning of taxa to the ASVs. Briefly, the forward and reverse reads upon filtering were set to a precautionary minimum length of 50 bp to avoid any possible low-quality reads as an alternative to read-end truncation. Read-end truncation was avoided due to high within-species ITS sequence length variations. Maximum expected error rate in the algorithm was set at 2 and all “N” nucleotides forbidden. From an input of 6,512 unique sequences, which had been generated from 40,182 total sequences read, the pipeline generated 92 true sequence variants. The denoised forward and reverse reads were then merged and a sequence table matrix, composed of sample IDs (rows) and ASVs (columns), constructed. Chimeric sequences were then removed to exclude artifact fungal sequences. The chimeras comprised 10% of merged sequence reads, giving an acceptable final 90% recovery of the raw input sequence variants. Taxonomy was then assigned to the generated ASVs by naive Bayesian classifier using the UNITE ITS fungal database (version 8.0 of 2019) (54). The generated sequence table matrix was then processed in Phyloseq (55) for the data analysis.

### Mycotoxins extractions and chromatographic analysis.

All mycotoxin extractions were liquid phase and analyte purification achieved using immunoaffinity columns (IACs). All purified analyte (AF and FB1) separations by high-pressure liquid chromatography (HPLC) were reversed phase through a 4.6-mm internal diameter (i.d.) × 100-mm length × 3.5-μm particle size Eclipse C_18_ column (Zorbax; Agilent Technologies, Santa Clara, CA, USA). The HPLC used was an Agilent Infinity II 1260 Series (Agilent Technologies, CA, USA) equipped with a fluorescence detector (FLD) and an autosampler for sample injections. AF-B1, AF-G1, and FB1 were derivatized to enable their detection by FLD. The AF and FB1 concentration data was acquired and generated using the Agilent ChemStation software prior to the data analysis. The quantification was by matrix-assisted calibration curve based on AF/FB1 fluorescence peak area (as response factor) and corresponding spiked-AF/FB1 concentration (as known variable).

### (i) Aflatoxin.

For season-1, a 50-g sample portion was extracted according to the standard AflaTest Vicam method for determination of aflatoxins by high-pressure liquid chromatography (HPLC) (www.vicam.com). The blending was done using a 1-L blender (Waring Commercial, Stamford, CT, USA). Prior to injection of eluate into HPLC, eluted contents in a 3-mL glass vial were derivatized as follows: eluate was dried under a stream of nitrogen at 50°C in a sample concentrator workstation (part number 133718/09; Biotage, Uppsala, Sweden). The residue was then reconstituted by adding 200 μL acetonitrile/water 86:14 (vol/vol) and vortexed for 7 s. Next, 300 μL 70:20:10 (vol/vol) mixture of water/trifluoroacetic acid/glacial acetic acid was added to the 200 μL reconstituted material and vortexed for 3 s to mix. The mixture was allowed to react for 25 min at 65°C in the sample concentrator water bath (without streaming nitrogen), with glass tube covered with parafilm. The contents were cooled while covered in light protective foil and then 500 μL deionised water added. Mixture was then vortexed for 3 s then filtered (0.22 μm, Ø 13 mm, nylon) into a 2-mL screwcap glass HPLC amber vial. Immediately, 50 μL was injected by autosampler into HPLC. HPLC conditions are detailed in Data Set S7 in the supplemental material.

For season-2 the standard AOZ Vicam method for simultaneous extraction of aflatoxins, ochratoxins, and zearalenone was used. Briefly, 5 g homogenized sample portion was weighed into a 50-mL polypropylene tube, and 20 mL of 3:2 (vol/vol) acetonitrile/water was added. Tubes were shaken at amplitude 12 for 30 min on a shaker (Burrell Wrist Action shaker, model 75; Burrell Scientific, LLC, Pittsburgh, PA, USA). Tubes were then centrifuged at 6,000 × *g* for 2 min, and then 8 mL of the supernatant was diluted with 32 mL 0.01% Tween phosphate-buffered saline (T-PBS), pH 7.0 (0.01%). Next, 10 mL diluted extract (unfiltered) was immediately passed through an AOZ immunoaffinity column. The column was first washed with 10 mL 0.01% T-PBS, followed by 10 mL Milli-Q water, allowing airflow through the IAC bed prior to elution. The AF was eluted with 1.5 mL liquid chromatography-mass spectrometry (LC-MS) grade MeOH followed by a further 1.5 mL 0.1% acetic acid. The eluate was vortexed briefly to mix and was filtered (0.22 μm, nylon) into a 2-mL screw-cap glass amber HPLC vial for the HPLC analysis. Next, 50 μL was injected by autosampler into HPLC. The AF-B1 and AF-G1 were derivatized post-column-automated with an UVE photochemical reactor (part № 10617, LCTech, Bayern, Germany).

### (ii) Fumonisin-B1.

For both maize cropping seasons, extraction was done according to the standard Vicam method for determination of fumonisins in maize by HPLC (https://vicam.com/products/fumonitest). For the extraction, a 5-g sample was weighed into a 50-mL polypropylene tube and all reagent proportions maintained. In place of blending, tubes were shaken for 30 min at amplitude 12 on a shaker (Burrell Wrist Action shaker, model 75; Burrell Scientific, LLC, Pittsburgh, PA, USA). The contents were centrifuged at 6,000 × *g* for 2 min, and then 8 mL of the supernatant was diluted with 32 mL phosphate-buffered saline (PBS), pH 7.0, and mixed well as described in standard protocol. For the derivatization of the eluted extract, the eluate was evaporated to dryness at 50°C under a stream of nitrogen in a sample concentration workstation (part number 133718/09; Biotage, Uppsala, Sweden). The residue was redissolved with 200 μL 1:1 methanol/water. Next, a 60-μL portion of reconstituted material was transferred into a 2-mL screw-cap glass HPLC amber vial containing 540 μL *o*-phthaldialdehyde reagent (Vicam parts number G5004 and G5003, mixed). The mixture was vortexed for 30 s and allowed to stand for a further 1 min for the derivatization reaction. Immediately 80 μL was injected by autosampler into the HPLC. Elution was done in isocratic mode at a flow rate of 0.8 mL/min. The mobile phase constituted methanol/0.1 M sodium dihydrogen phosphate (23:77, vol/vol) buffered to pH 3.5 with *o*-phosphoric acid (detailed HPLC conditions in Data Set S7 in the supplemental material).

### Data analysis.

All statistical computations were conducted in software R version 4.1.0 (56) using the below packages and their functions:

Phyloseq (55) was used to generate the ASV relative abundances of fungal genera. Vegan (“Vegetation analysis”) (57) was used for computation of taxa diversity and composition. The package ggplot2 (58) was deployed for visualizations.

### (i) Fungal genera abundance/composition.

Seasonal fungal composition over the weather variables was evaluated using pairwise adonis2, with Bonferroni correction of *P* value. Permutational multivariate analysis of variance (PERMANOVA) was used for analysis of the ecological data (species composition) due to highly heterogenous distribution of such data (59). Fungal diversity was determined over the weather variables (S1, low rainfall with dry spell; S2, low rainfall; N1, high rainfall; N2, high rainfall) using the Shannon diversity measure. This was on the basis of the precision of the inferred species across the four variables (S1, S2, N1, and N2). Differences in computed fungal alpha diversity across the weather variables were evaluated by analysis of variance (ANOVA) (applied for the normal distributed alpha diversity data). The Wilcoxon rank-sum test was used to determine changes in fungal genera abundances between weather variables for nonnormal distribution or ANOVA for normal distribution data. Similarly, for comparisons of specific genera between AEZ, the Kruskal-Wallis rank sum test was used for nonnormal distributions (Shapiro Wilk test, *P* < 0.05) and *t* test for normal distributions. To compare *Aspergillus* relative abundance/quantity between HDSeq (%) and dilution plating (CFU/g), Spearman's rank correlation was used. This is given that the two methods did not produce the same abundance measurement but were rather expected to produce a good level of positive correlation. For example, the expectation is that absence of *Aspergillus* by HDSeq should show an absence of the *Aspergillus* by plating.

### (ii) Mycobiome composition in relation to weather variables.

The weather factor considered in the test was rainfall. The dependent variable, genera relative abundance, was computed against the four weather variables S1 (low rainfall with dry spell), S2 (low rainfall), N1 (high rainfall), and N2 (high rainfall).

### (iii) Correlation between *Aspergillus*/*Fusarium* and maize aflatoxin/fumonisin-B1.

Spearman’s rank correlation rho was used to determine correlation between genus *Aspergillus* and AF as well as *Fusarium* and FB1.

### Data availability.

DNA sequences of the fungal amplicon sequence variants (ASVs) were deposited into GenBank. Code for mycobiome census as well as GenBank accession numbers for the ASVs (OQ645348 - OQ645435; OQ683600 - OQ683780) are found at https://github.com/bkatati/mmycobiome. Additional data is available upon request from the corresponding authors.
