# Supplementary material for: Preharvest Maize Fungal Microbiome and Mycotoxin Contamination: Case of Zambia’s Different Rainfall Patterns
Source: Appl Environ Microbiol. 2023 May 31;89(6):e00078-23. doi: 10.1128/aem.00078-23 (PMC10304879; doi:10.1128/aem.00078-23)
Supplement: Supplemental file 1 — Supplemental material. Download aem.00078-23-s0001.docx, DOCX file, 0.04 MB [file aem.00078-23-s0001.docx]

**Data Set S1**. Maize moisture percentage in maize samples at harvest.

| **Field** | **% (S1)** |  | **Field** | **% (N1)** |  | **Field** | **% (S2)** |  | **Field** | **% (N2)** |
| --- | --- | --- | --- | --- | --- | --- | --- | --- | --- | --- |
| 1-01 | 34.0 |  | 1-21 | 15.7 |  | 3-41 | 28.0 |  | 3-61 | 14.6 |
| 1-02 | 25.1 |  | 1-22 | 15.4 |  | 3-42 | 17.5 |  | 3-62 | 14.1 |
| 1-03 | 28.0 |  | 1-23 | 15.0 |  | 3-43 | 20.7 |  | 3-63 | 18.1 |
| 1-04 | 20.2 |  | 1-24 | 17.4 |  | 3-44 | 17.8 |  | 3-64 | 18.8 |
| 1-05 | 25.2 |  | 1-25 | 13.5 |  | 3-45 | 22.5 |  | 3-65 | 19.9 |
| 1-06 | 29.7 |  | 1-26 | 13.2 |  | 3-46 | 22.7 |  | 3-66 | 19.2 |
| 1-07 | 15.8 |  | 1-27 | 19.0 |  | 3-47 | 15.6 |  | 3-67 | 17.8 |
| 1-08 | 24.8 |  | 1-28 | 17.1 |  | 3-48 | 13.1 |  | 3-68 | 14.9 |
| 1-09 | 23.2 |  | 1-29 | 17.5 |  | 3-49 | 22.7 |  | 3-69 | 18.8 |
| 1-10 | 21.5 |  | 1-30 | 14.3 |  | 3-50 | 31.9 |  | 3-70 | 14.3 |
| 1-11 | 34.0 |  | 1-31 | 13.6 |  | 3-51 | 12.9 |  | 3-71 | 16.2 |
| 1-12 | 34.5 |  | 1-32 | 13.0 |  | 3-52 | 24.8 |  | 3-72 | 15.1 |
| 1-13 | 19.3 |  | 1-33 | 15.8 |  | 3-53 | 22.8 |  | 3-73 | 18.4 |
| 1-14 | 22.9 |  | 1-34 | 13.8 |  | 3-54 | 18.1 |  | 3-74 | 20.5 |
| 1-15 | 17.9 |  | 1-35 | 15.7 |  | 3-55 | 18.2 |  | 3-75 | 17.6 |
| 1-16 | 25.3 |  | 1-36 | 13.6 |  | 3-56 | 13.5 |  | 3-76 | 14.4 |
| 1-17 | 17.0 |  | 1-37 | 13.5 |  | 3-57 | 14.2 |  | 3-77 | 22.5 |
| 1-18 | 21.0 |  | 1-38 | 13.1 |  | 3-58 | 33.1 |  | 3-78 | 15.2 |
| 1-19 | 14.6 |  | 1-39 | 11.5 |  | 3-59 | 21.4 |  | 3-79 | 17.3 |
| 1-20 | 17.1 |  | 1-40 | 15.1 |  | 3-60 | 14.6 |  | 3-80 | 14.5 |
| Average | *23.6* |  | Average | *14.8* |  | Average | *20.3* |  | Average | *17.1* |
| Std_Dev | *6.1* |  | Std_Dev | *1.9* |  | Std_Dev | *5.9* |  | Std_Dev | *2.4* |
| Min | *14.6* |  | Min | *11.5* |  | Min | *12.9* |  | Min | *14.1* |
| Max | *34.5* |  | Max | *19.0* |  | Max | *33.1* |  | Max | *22.5* |

**Data Set S2.**

Very low frequency and abundance genera - Genera appearing only in one field out of the 80 samplings with relative abundance < 0.1%, (n = 24) were as follows: *Articulospora, Ascochyta, Chaetomium, Chalara, Clonostachys, Colacogloea, Coniothyrium, Cryptococcus, Cyanodermella, Cyphellophora, EuPenicillium, Exophiala, Kwoniella, Melampsora, Neoascochyta, Olpitrichum, Periconia, Phaeosphaeria, Pseudopithomyces, Pyricularia, Saturnispora, Simplicillium, Sphaeronaemella, Tremella*. Due to their very low relative abundance and frequency, it could not be completely ascertained if they belonged to the mycobiome.

**Data Set S3**. Evaluation of Loss of Viable Fungal Mass during Kernel Wash Pelleting.

The protocol described in materials and methods for generation of pellet of mycobiome DNA extraction was followed. It was evaluated for extent of loss of viable fungal spores during the multiple washing steps to wash off the surfactant, Triton-X. Maize kernels were weighed into a 250 ml flask and spores extracted by shaking in 0.05% triton-X at concentration 1g kernels /ml as described in the protocol under materials and methods section. The first supernatant of the kernel wash from the first centrifuge step (materials and methods section) was pipetted off into a 1.5 ml microcentrifuge tube for plating, and assigned 1^st^ supernatant. The remaining ≈ 2 ml lower fraction was transferred to a microcentrifuge tube, as described in materials and methods, and centrifuged. About 500 µl of the second supernatant was transferred to a microcentrifuge tube for plating and assigned 2^nd^ supernatant. The remaining lower fraction of approximately 500 µl was assigned 2^nd^ lower fraction. It was pipetted off from the pellet into another 1.5 ml microcentrifuge tube prior to addition of further washing water (MQ sterile water) to the pellet. There was no 1^st^ lower fraction as all lower fraction was transferred to microcentrifuge tubes. A total of four supernatants and three lower fractions were therefore prepared and plated by the dilution plate technique on 90 mm petri dishes with PDA media (7 days, 25 °C, dark). The quantity of spores (Q, spores) was determined as product of volume of suspension (V, ml) x concentration of spores (C, sp/ml) in the suspension (Supplemental Table 2). The concentration of the spores had been determined by spore count from the PDA plates.

**Data Set S4**. Estimation of viable fungal spore loss while pelleting kernel surface wash.

| Fraction | Sample1 | | | | Sample2 | | |
| --- | --- | --- | --- | --- | --- | --- | --- |
|  | C, sp/ml | V, ml | Q, sp | C, sp/ml | | V, ml | Q, sp |
| 1st supernatant | 0.0 | 31.0 | 0.00 | 0.0 | | 35.0 | 0.00 |
| 2nd supernatant | 0.0 | 0.5 | 0.00 | 0.0 | | 0.4 | 0.00 |
| 2nd lower fraction | 0.0 | 0.5 | 0.00 | 3.3 | | 0.4 | 1.35 |
| 3rd supernatant | 0.0 | 0.5 | 0.00 | 0.0 | | 0.4 | 0.00 |
| 3rd lower fraction | 0.0 | 0.5 | 0.00 | 0.0 | | 0.4 | 0.00 |
| 4th supernatant | 0.0 | 0.5 | 0.00 | 3.3 | | 0.4 | 1.35 |
| 4th lower fraction | 3.3 | 0.5 | 1.65 | 10.0 | | 0.4 | 4.05 |
| Total  (lost spores): |  |  | 1,65 |  | |  | 6,75 |
| Pellet (spores): | 2000 | 0.48 | 960 | 2833 | | 0.72 | 2040 |
| % spore loss: |  |  | 0.17 |  | |  | 0.33 |

**Data Set S4** shows the quantity of spores (Q) per fraction in centrifuge tube. C = spore concentration per fraction. V = approximate volume of fraction in tube. Q (spore per fraction) = C x V. Total spore loss from pellet = 0.25 ± 0.11 %.

**Data Set S5**. DNA recovery in mock fungal community

| **Pre-sequencing spike ratio, %** | | **post ASV clean-up abundance, %** | | **Std. deviation** | **Recovery, %** | |
| --- | --- | --- | --- | --- | --- | --- |
| *Aspergillus* | *Fusarium* | *Aspergillus* | *Fusarium* |  | *Aspergillus* | *Fusarium* |
| 5 | 95 | 5.1 | 94.9 | ± 0.2 | 101.1 | 99.9 |
| 15 | 85 | 16.5 | 83.5 | ± 0.0 | 110.2 | 98.2 |
| 50 | 50 | 48.0 | 52.0 | ± 1.7 | 96.0 | 103.9 |

**Data Set S5** shows Amplicons Sequence Variant recovery of spiked DNA of *Fusarium* *verticillioides* and *Aspergillus* *flavus* as mock community.

**Data Set S6** shows comparison of *Aspergillus* abundance on maize kernels by high-throughput DNA amplicon Sequencing (HDSeq) and plating. Fields (samples) shown had *Aspergillus* detected by HDSeq only or HDSeq and plating. All fields positive for *Aspergillus* by plating were positive by sequencing. Number of fields positive for *Aspergillus*: Sequencing = 38/80; Plating = 19/80. Fields without *Aspergillus* by both methods are not shown. Fields 1 – 20 = Season1, AEZ1 (S1, low rainfall with dry spell); Fields 21 – 40 = Season1, AEZ3 (N1, high rainfall); Fields 41 – 60 = Season2, AEZ1 (S2, low rainfall); Fields 61 – 80 = Season2, AEZ3 (N2, high rainfall).

**Data Set S7.** HPLC conditions for mycotoxins detection

| **Analyte** | Mycotoxin | Aflatoxin | Aflatoxin | Fumonisin |
| --- | --- | --- | --- | --- |
| **Method** | ™ | Vicam AOZ | RomarLabs WB | Vicam FumoniTest WB |
| **Mobile phase** | v/v | Methanol:water 45:55 | Acetonitrile:water 20:80 | Methanol:0.1M sodium dihydrogen phosphate (23:77) |
|  | Flow rate, ml/min | 0.5 | 0.8 | 0.8 |
| **Column oven** | °C | 30 | | 30 |
|  |  |  | |  |
| **Detector** | Excitation, nm | 365 | | 335 |
|  | Emission, nm | 455 | | 440 |
| **Matrix calibrants** | µg/kg | 1, 5, 10, 50, 100 and 500 | | 7.5, 15, 30, 60, 150 and 250 |
| **Limit of Detection** | **µg/kg** | B_1_ = 0.29; B_2_ = 0.22; G1 = 0.30; G_2_ = 0.28 | | 1.0 |

**Data Set S7** shows Instrument conditions for the acquisition and detection of aflatoxins-B1, -B2, -G1, -G2 and fumonisin-B1 in maize.

**Data Set S8A )** Aflatoxin and fumonisin levels in pre-harvest maize.

| **Field #** | **Season** | **AEZ** | **Weather Variable** | **District** | **AF-B1 range, µg/kg** | **Total, AF, range µg/kg** | **FB1 ^ɨ^ range, µg/kg** |
| --- | --- | --- | --- | --- | --- | --- | --- |
| 1 – 5 | One | I | S1 | Kalomo  ^(*= x1; ꬷ = x1)^ | 0.9 – 33.9 | 13.5 | 18.7 - 357.4 |
| 6 - 10 | One | I | S1 | Kazungula  ^(*= x1; ♯ = x1)^ | 0.9 - 118.2 | 1.7 - 208.2 | 32.1 - 938.3 |
| 11-15 | One | I | S1 | Livingstone  ^(ꬷ = x1; ♯ = x1 ; * = x1; y =1)^ | 1.1 – 306.8 | 1.9 – 348.4 | 15.1 – 1787.1 |
| 16-20 | One | I | S1 | Mulobezi  ^(ꬷ = x1 ; * = x1; y = x1; z =1)^ | 0.9 – 35.8 | 1.7 – 58.7 | 21.4 - 1715.9 |
| 21-25 | One | III | N1 | Samfya | ND | ND | 20.9 - 493.9 |
| 26-30 | One | III | N1 | Mansa | ND | ND | 12.6 - 488.1 |
| 31-35 | One | III | N1 | Luwingu  ^(z =1)^ | ND | ND | 28.7 - 1454.9 |
| 36-40 | One | III | N1 | Kawambwa  ^(y = x1)^ | ND | ND | 11.4 - 1487.0 |
| 41-45 | Two | I | S2 | Kalomo  ^(y = x1; z =1)^ | ND | ND | 1.0 - 4328.5 |
| 46-50 | Two | I | S2 | Kazungula  ^(y = 2; z =1)^ | ND | ND | 1.0 - 4764.8 |
| 51-55 | Two | I | S2 | Livingstone  ^(y = x1)^ | ND | ND | 1.0 - 3681.5 |
| 56-60 | Two | I | S2 | Mulobezi | ND | ND | 1.0 - 962.2 |
| 61-65 | Two | III | N2 | Samfya  ^(y = x1)^ | ND | ND | 1.0 - 1694.8 |
| 66-70 | Two | III | N2 | Mansa | ND | ND | 1.0 – 51.1 |
| 71-75 | Two | III | N2 | Luwingu | ND | ND | 1.0-1.0 |
| 76-80 | Two | III | N2 | Kawambwa | ND | ND | 1.0 - 109.5 |

a) shows district fields aflatoxin AF and FB1 levels in pre-harvest maize. ^(*)^ = 10 – 50 µg/kg; ^(ꬷ)^ = 50 – 100 µg/kg; ^(♯)^ > 100 µg/kg. ND = limit of detection: AF-B1 = 0.29 µg/kg, Total AF = 1.09, µg/kg as sum of detection limits for AF-B1, -B2, -G1 and -G2.

^ɨ^ FB1 adjusted value by multiplication factor x1.6 taking into consideration analytical recovery value for FB1 of 60% based on value of reference material extracted alongside samples; ^(y)^ = FB1 > 1000 µg/kg (EU regulatory limit); ^(z)^ = FB1 > 4000 µg/kg (US Food and Drug regulatory limit). ND FB1 = 1 µg/kg**.**

**Data Set S8B)** shows geometric mean values of aflatoxin and Fumonisin-B1.

|  |  | S1 | N1 | S2 | N2 |
| --- | --- | --- | --- | --- | --- |
| Fumonisin-B1 | Geo_Mean | 116.9 | 63.5 | 60.2 | 3.2 |
|  | Range, µg/kg | 9.5 - 1116.9 | 7.2 - 929.4 | 1 – 2978.0 | 1 – 1059.2 |
| Aflatoxin | Geo_Mean, | 6.7 | 0 | 0 | 0 |
|  | Range, µg/kg | 0 - 352 | 0 | 0 | 0 |

Weather patterns: S1 = ‘Dry spell’; N1 = “High rainfall”, S2 = “Moderate rainfall”, N2 = “High rainfall.” Levels of fumonisin-B1 were only significantly different between N2 and rest of weather variables (N2:N1, *P* < 0.001; N2:S1, *P* < 0.001; N2:S2, *P* < 0.001).

Levels of fumonisin-B1 were significantly higher in the southern agroecology (AEZ1) as a whole compared to northern agroecology (AEZ3). FB1, Ln µg/kg : AEZ1 (4.4) > AEZ3 (2.7), (T-test, *P* = 0.002). S1 had significantly higher fumonisin-B1 than N2 (pairwise t-test *P* = 0.014).
